# Musculoskeletal anomalies in a national cohort of children and adolescents with trisomy 21

**DOI:** 10.1186/1546-0096-12-S1-P160

**Published:** 2014-09-17

**Authors:** Charlene Foley, Emma MacDermott, Orla Killeen

**Affiliations:** 1 Rheumatology, National Centre for Paediatric Rheumatology (NCPR), Dublin, Ireland

## Introduction

Musculoskeletal complications of Down syndrome are common. Joint laxity, which may be associated with delayed ambulation, is almost universal. The combination of this ligamentous laxity and low muscle tone contribute to an increased risk of a number of musculoskeletal disorders in children with Down syndrome.

## Objectives

1. To describe the musculoskeletal anomalies observed in a National cohort of children with tisomy 21.

2. To calculate the average age children with Down syndrome walked unaided in our cohort.

3. To determine if in particular, musculoskeletal disorders in Down syndrome pose a greater problem for teenagers than children.

## Methods

Over a 6-month period we performed a musculoskeletal examination on children and young adults with Down syndrome. We documented their Beighton hypermobility score, and any relevant orthopaedic history.

## Results

198 children and adolescents were examined, (56% male, 44% female). Median age was 7.2 years (0.6-18.6 years); 26% were adolescents (i.e. age ≥ 12 years). The average Beighton score for adolescents was 2 (0-6), which was significantly lower than that recorded for children, 4 (0-9) (p<0.001). There was no significant difference in the number of orthopedic conditions observed in children (10%) and adolescents (16%). They included scoliosis, patella instability, hip dislocation and C-spine anomalies.

Pes planus was seen in 67% of children and 80% of adolescents with Down syndrome. However, only 42% of these children and 51% of the adolescents wore orthotics. The median age our cohort walked was 28 months (12-84 months). This is comparable to the literature that reports children with Down syndrome walk at 23 months (range 13-48), compared with 12 months (range 9-17) for the general paediatric population.

## Conclusion

- Pes planus is common in children with Down syndrome, therefore early consideration of orthotics and life-long appropriate supportive footwear is advised.

- Significantly delayed ambulation is noted in children with Down syndrome. Early multi-disciplinary intervention is important to ensure these children obtain their full potential with regards to acquisition of walking unaided.

- Children with Down syndrome are at increased risk of a number of potentially debilitating orthopaedic conditions in addition to C-spine instability.

- Hypermobility becomes a less prominent musculoskeletal feature in the adolescent with Down syndrome.

## Disclosure of interest

None declared.

